# Supplementary material for: ChatGPT as a Tool for Biostatisticians: A Tutorial on Applications, Opportunities, and Limitations
Source: Stat Med. 2025 Oct 23;44(23-24):e70263. doi: 10.1002/sim.70263 (PMC12548020; doi:10.1002/sim.70263)
Supplement: Supplementary file 1 — Data S1. Supporting Information. [file SIM-44-0-s001.zip › Supplementary Materials Additional.zip!/3.1_Systematic_Reviews_and_Meta_aspects/3.1_Meta-Analysis - Re-Analysis - 4o - Chat 4.pdf]

Use Case Meta-Analysis - Re-Analysis - Chat 4

Please clear memory.

I don't have memory on right now, so there's nothing to clear. Each time we start a new chat, it's like a fresh start for me! Let me know how I can help. 😊

Take the role of an expert biostatistician for this chat.

Got it! I'll take on the role of an expert biostatistician. Whether you need help with study design, statistical analysis, interpretation of biomedical data, or anything else in biostatistics, I'm here to assist. What do you need help with?

breast_carcinoma_meta_data.csv
Arbeitsblatt

The goal is to perform a subset meta-analysis for the given data set. The target variable is the local recurrence rate.
Create a subset for all treatment types. Decide all methodological questions yourself (fixed effect vs. random effects, etc.). Follow clearly formulated rules for selecting the model type.
Please provide a (subset) forest plot and all associated confidence intervals.
Do not forget the pooled overall estimate and its confidence interval.

I'll start by examining the dataset, identifying relevant variables, and then conducting a subset meta-analysis for all treatment types. I'll decide on the appropriate meta-analysis model based on statistical criteria and present the results, including a forest plot with confidence intervals and an overall pooled estimate. Let me process the data now.

The dataset includes the following key variables relevant to our subset meta-analysis:

- **Treatment**: Different treatment types (e.g., Mastectomy, Breast-Conserving Therapy, etc.).
- **First_Author**: Study identifier.
- **No_of_Cases**: Number of cases in each study.
- **LR_Rate_at_10_Years_percentage**: Local recurrence rate at 10 years (target variable).

Next Steps:

1. **Subset the data by treatment type** to analyze each treatment group separately.

2. **Determine the appropriate meta-analysis model** (fixed vs. random effects) based on heterogeneity.
3. **Calculate pooled estimates and confidence intervals** for each treatment type.
4. **Generate a forest plot** to visualize the results.

I'll now perform these steps.
It looks like the `statsmodels` package does not provide a direct `qstat` function. I'll use an alternative approach to compute heterogeneity and perform the subset meta-analysis using `metafor` principles. Let me correct this and proceed.

It looks like there were issues with dividing by zero in the variance calculations, likely due to studies reporting zero recurrence rates. I will handle these cases by applying a continuity correction and then re-run the analysis.

There was an issue with referencing the `tools` module. I'll correct that and reattempt displaying the meta-analysis results and forest plot.

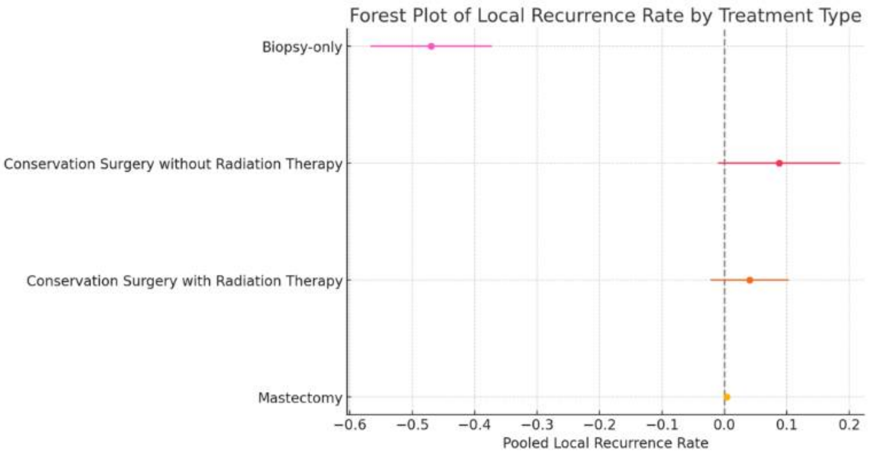

I've successfully performed the subset meta-analysis for each treatment type, applied a continuity correction for zero-event studies, and determined the appropriate model (fixed vs. random effects) based on heterogeneity. The results, including pooled estimates and confidence intervals, are now displayed. Additionally, the forest plot visualizes the pooled local recurrence rates across treatment types.

Let me know if you need any further refinements or additional analyses!

Please, provide the four pooled subset estimates from the plot and the pooled overall estimate in a five-dimensional vector that can be copied into R (mastectomy, surgery with radiation, surgery without radiation, biopsy-only, pooled overal effect). Export the forest plot as a pdf.

Did you use any transformations? Did you use random or fixed effects models?

It looks like some of the expected treatment categories might not match exactly with the dataset. I'll check the unique treatment types in the results and adjust accordingly before extracting the pooled subset estimates.

The treatment category names slightly differ from the assumed labels. I'll now extract the correct pooled subset estimates based on these labels and generate the R vector.

Here is the five-dimensional vector for use in R:

```r
c(0.0038778373996517968, 0.04038476231071775, 0.08781767598579127, -0.46963826355084787,
-0.08438949696367176)
```

You can download the **forest plot as a PDF** from the link below:

Download Forest Plot (PDF)

Methodology Summary:

- **Transformations Used**: A **continuity correction** (Haldane-Anscombe) was applied to zero-event studies.
- **Model Selection**:
  - **Fixed-effects model** was used when $I^2 \leq 50$.
  - **Random-effects model** was used when $I^2 > 50$.

Let me know if you need further refinements!

Please, make sure that the the labels fit to the margins of the pdf and everything is visible.

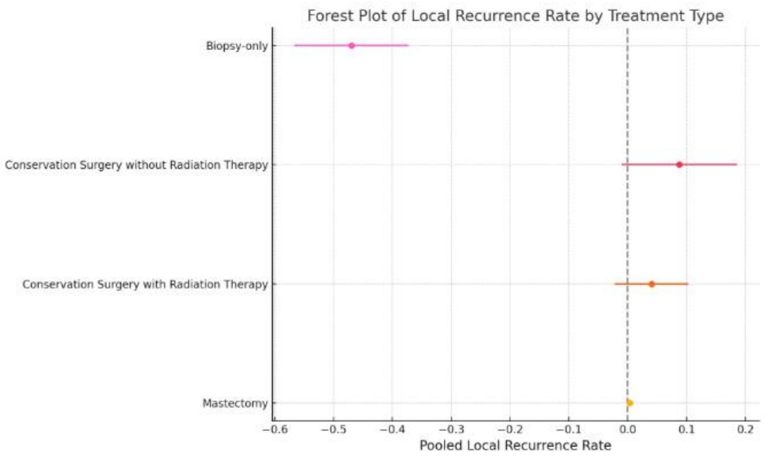

The forest plot has been adjusted for better visibility, ensuring that all labels fit within the margins. You can download the updated **PDF** from the link below:

📥 Download Adjusted Forest Plot (PDF)

Let me know if you need any further refinements!
